# Supplementary material for: Virus Yellows and Syndrome “Basses Richesses” in Western Switzerland: A Dramatic 2020 Season Calls for Urgent Control Measures
Source: Pathogens. 2022 Aug 6;11(8):885. doi: 10.3390/pathogens11080885 (PMC9414692; doi:10.3390/pathogens11080885)
Supplement: Supplementary file 1 [file pathogens-11-00885-s001.zip › Supplementary_File1_v2.pdf]

Supplementary tables.

Table S1. Screening of VY viruses *Ca.* A. phytopathogenicus in samples of sugar beet from different locations in Switzerland.

Sites	BYV	BMYV	BChV	BWYV	BtMV	*Ca.* A. phytopathogenicus
**West**						
Alle	4/4	0/4	2/4	0/4	0/4	-
Bargen	0/1	0/1	1/1	0/1	0/1	-
Bavois	2/2	0/2	2/2	0/2	0/2	-
Büetigen	2/3	0/3	2/3	0/3	0/3	0/3
Changins	3/3	0/3	3/3	0/3	0/3	2/3
Chavornay	3/3	0/3	3/3	0/3	0/3	3/3
Fräschels	9/9	0/9	7/9	0/9	0/9	4/9
Gals	7/8	2/8	8/8	0/8	0/8	1/3
Ins	7/9	0/9	8/9	0/9	0/9	1/2
Kappelen	0/3	0/3	2/3	0/3	0/3	1/3
Kiesen	0/3	0/3	3/3	0/3	0/3	0/3
Leuzingen	3/3	0/3	3/3	0/3	0/3	1/3
Lyssach	2/3	0/3	2/3	0/3	0/3	-
Marnand	3/3	0/3	1/3	0/3	0/3	3/3
Montignez	1/1	0/1	0/1	0/1	0/1	-
Moosseedorf	9/9	1/9	9/9	0/9	0/9	1/3
Payerne	3/3	0/3	3/3	0/3	0/3	3/3
Penthalaz	2/2	0/2	2/2	0/2	0/2	-
Seedorf	1/1	0/1	1/1	0/1	0/1	-
Suberg	0/3	0/3	3/3	0/3	0/3	1/3
Vicques	3/4	0/4	2/4	0/4	0/4	-
**Total**	**64/80**	**3/80**	**67/80**	**0/80**	**0/80**	**21/44**
**%**	**80.0**	**3.7**	**83.7**	**0**	**0**	**47.7**
**East**						
Altikon	0/2	0/2	2/2	0/2	0/2	0/2
Andelfingen	0/2	0/2	1/2	0/2	0/2	0/1
Dachsen	2/2	0/2	0/2	0/2	0/2	0/1
Ellikon	0/1	0/1	0/1	0/1	0/1	0/1
Endingen	1/1	0/1	0/1	0/1	0/1	0/1
Felben	0/1	0/1	1/1	0/1	0/1	0/1
Frauenfeld	0/2	0/2	1/2	0/2	0/2	0/2
Haag	0/2	0/2	2/2	0/2	0/2	0/1
Hagenbuch	0/1	0/1	0/1	0/1	0/1	0/1
Marthalen	1/1	0/1	0/1	0/1	0/1	-
Oetwil an der Limmat	1/1	0/1	0/1	0/1	0/1	-
Ramsen	1/1	0/1	0/1	0/1	0/1	0/1
Schleitheim	1/1	0/1	0/1	0/1	0/1	-
**Total**	**7/18**	**0/18**	**7/18**	**0/18**	**0/18**	**0/12**
**%**	**38.9**	**0**	**38.9**	**0**	**0**	**0**

Table S2. Details for the Illumina sequencing of Swiss isolates of BYV and BChV.

Tag	Origin	RT-PCR		Total reads	Mapped reads	
		BYV	BChV		BYV	BChV
11	Seedorf	+	+	878,784	19,771	89
13	Haag	-	+	1778,933	-	159
15	Bargen	-	+	898,120	-	982
18	Dachsen	+	-	940,195	803,589	-
44	Bavois1	+	+	682,276	557,890	99
78	Bavois2	+	+	580,241	538,613	57
93	Ramsen	+	-	137,415	105,230	-
95	Villiger	-	+	946,377	-	83

Table S3. Features of complete or near complete genomes of BYV, BChV and BMYV.

Virus isolate	Accession number	Size (nt)	Origin	Collection date
BYV-U	X73476.1	15480	Ukraine	1994
BYV-Ca	AF056575.1	15468	USA	1998
BYV-4	AF190581.1	15468	USA	1999
BYV-PV1260	MT815988.1	15469	Germany	2021*
BYV-PV1237	MT701720.1	15470	UK	2021*
BYV-PV0981	MW274719.1	15468	Unknown	2021*
BYV-Seedorf	ON738345	15470	Switzerland	2020
BYV-Dachsen	ON738343			
BYV-Bavois1	ON738341			
BYV-Bavois2	ON738342			
BYV-Ramsen	ON738344			
BChV-2a	AF352024.1	5776	UK	2002
BChV-CR	AF352025.1	5742	USA	2002
BChV- PV1211	MW367424.1	5744	France	2021*
BChV-MPTGP-ZA	MN734427.1	5773	South Africa	2016
BChV-Bargen	ON738346	5717	Switzerland	2020
BChV-Haag	ON738347	5753		
BChV-Villiger	ON738348	5754		
BMYV-2ITB	NC_003491.1	5722	France	1995
BMYV -EK	KC121026.1	5723	France	2008
BMYV-IPP	DQ132996.1	5723	Germany	2006
BMYV-Broom's Barn	EF107543.1	5721	UK	2006
BMYV-PV1210	MW367423.1	5653	Germany	2021*
BMYV-Gals1	ON738349	5688	Switzerland	2020
BMYV-Gals2	ON738350	5689		
BMYV-Moosseedorf	ON738351	5683		

*Corresponds to the uploading date. The actual collection date is not known.

Table S4. Compositions of the RT-PCR and PCR reaction mixes.

	**Volume in PCR mix (μl)**	**Volume in RT-PCR mix (μl)**
H_2O	13.3	11.95
5X flexi G2 green buffer	5	5
$MgCl_2$ (25mM)	3	3.5
dNTP (10mM each)	0.5	2
Forward primer (50mM)	0.5	0.5
Reverse primer (50mM)	0.5	0.5
GoTaq G2 (5u/μl)	0.2	0.25
RNasin (40u/μl)	-	0.15
AMV-RT (10u/μl)	-	0.15
Sample	2	1

Supplementary figures.

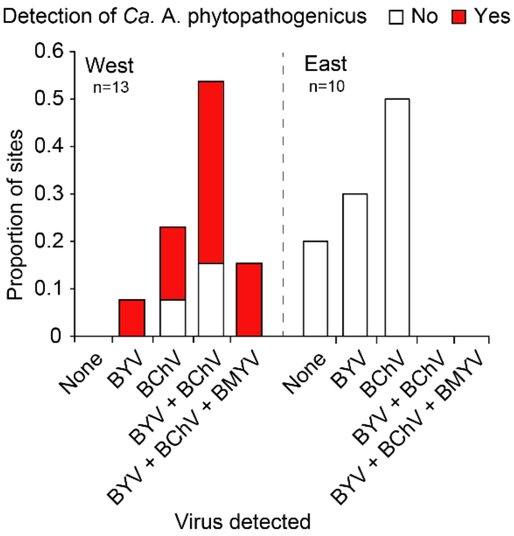

Figure S1. Proportion of sites positive for VY and SBR where causal agents for both diseases were screened.

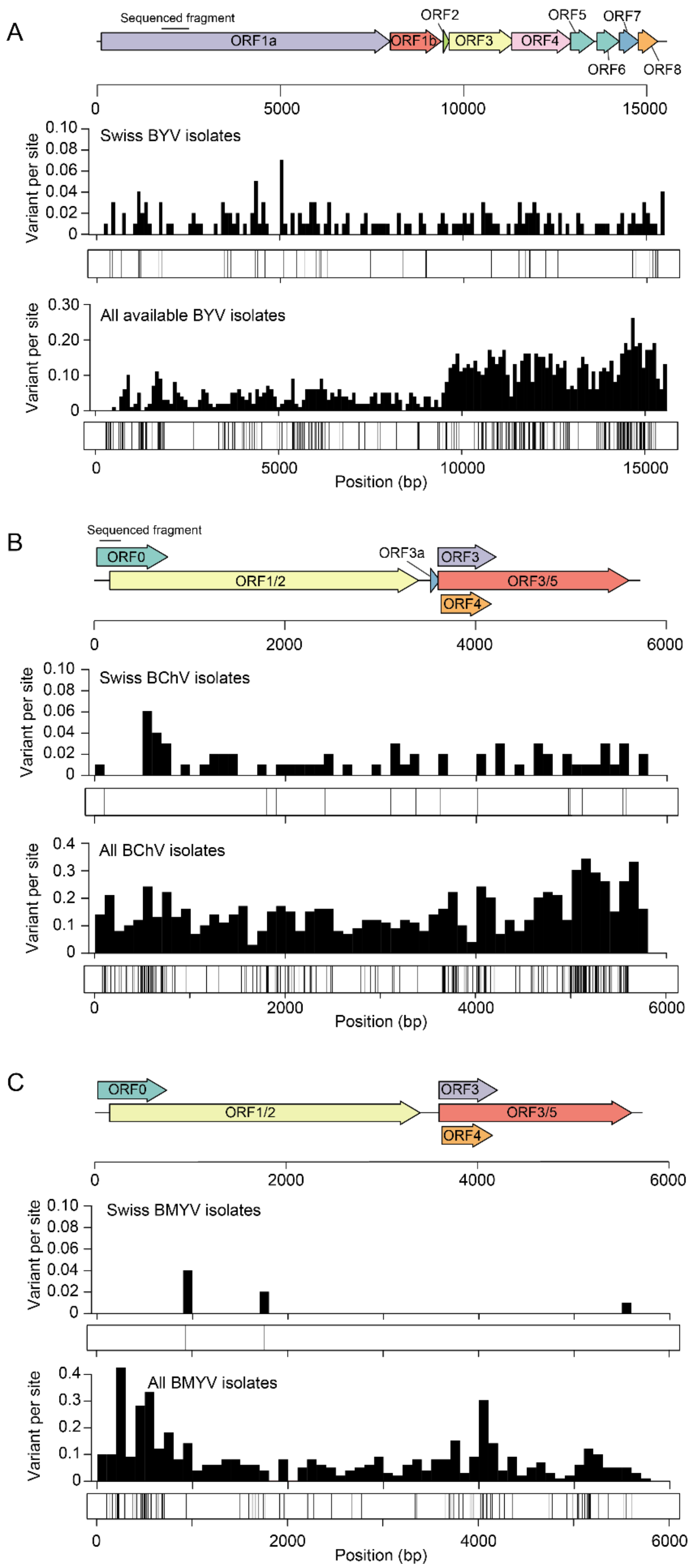

Figure S2. Sliding window analysis of BYV, BChV and BMYV genomes. The genetic diversity across BYV (A), BChV (B) and BMYV (C) sequences are shown for Swiss genomes (middle) or all available genomes (bottom). Window and step size are 100bp. The box under each sliding window analysis shows the location of non-synonymous mutations along the genome. Each line represents a single variant.

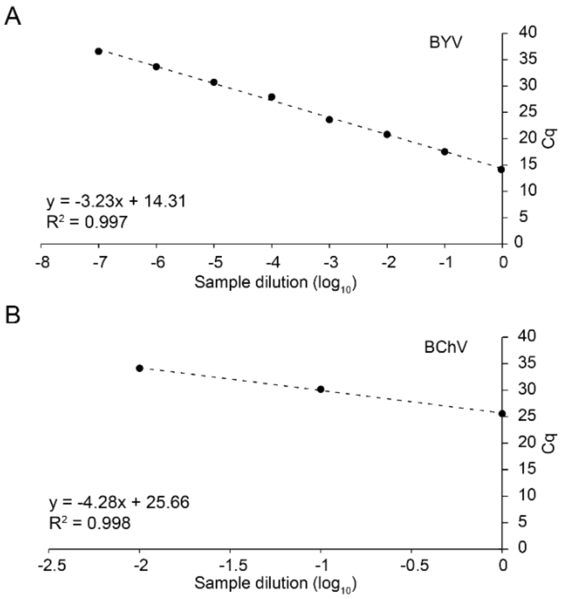

C

		BYV	BChV
**Duplex**	Efficiency	104%	71%
	Detection limit (Cq)	38.1	37.2
**Triplex**	Efficiency	108%	82%
	Detection limit (Cq)	32.1	33.2

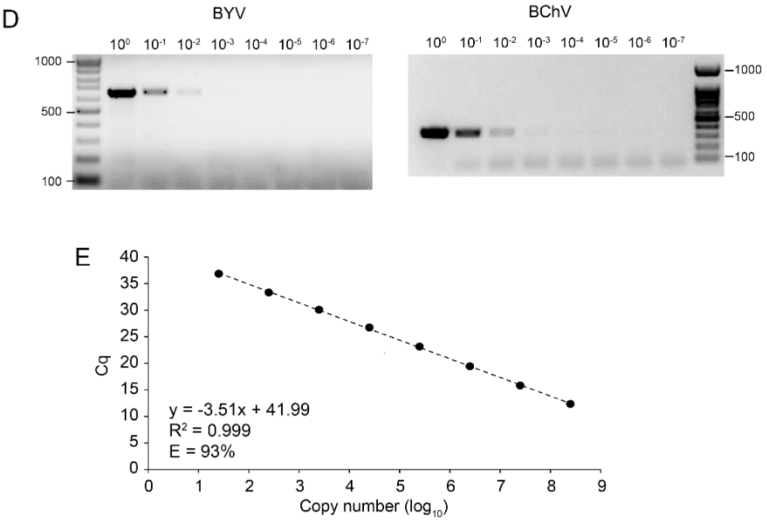

Figure S3. Evaluation of the novel (RT-)qPCR tools. (A and B) Cq values obtained by duplex RT-qPCR on RNA samples of sugar beet infected by BYV (A) or BChV (B) in serial dilutions in healthy beet extract. The average Cq value of technical triplicates is shown; (C) Efficiencies and detection limit for BYV and BChV in duplex and triplex RT-qPCR reactions. The detection limits were determined as the last dilution which gave a reliable signal in six technical replicates; (D) Determination of the detection limit of RT-PCR using previously published primers. The same diluted samples used to calibrate the duplex and triplex RT-qPCR (as in A and B) were used for comparison; (E) Cq values obtained by qPCR on a recombinant pGemT-Easy plasmid harboring the partial *Ca.* A. phytopathogenicus SpoT sequence, in serial dilution in healthy beet extract. The average Cq value of technical duplicates is shown.

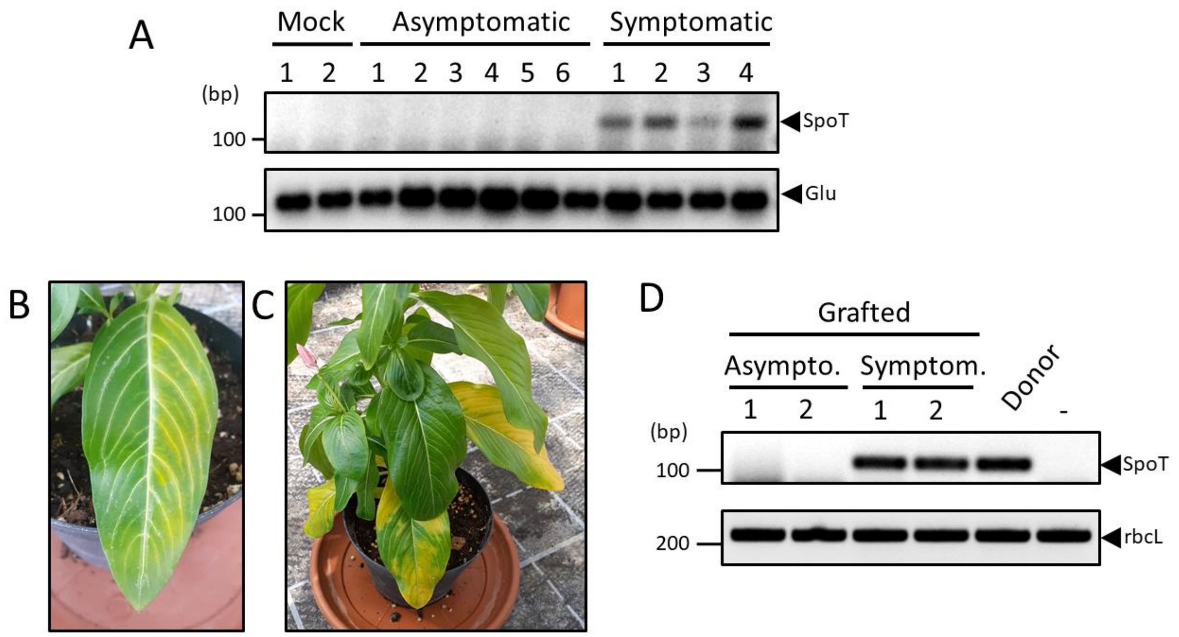

Figure S4. Detection of *Ca.* A. phytopathogenicus in inoculated sugar beet and Madagascar periwinkle. (A) Agarose gel electrophoresis of amplicons obtained by PCR analysis on sugar beet roots following insect-mediated inoculation at 90 dpi; (B) Vein yellowing on periwinkle leaf following insect-mediated inoculation at 40 dpi; (C) Same plant as in B at 50 dpi; (D) Agarose gel electrophoresis of PCR amplicons for the analysis of stem samples from four periwinkles grafted with a SBR-positive stem. Donor = insect-inoculated plant (shown in B and C) that was used to provide the infected stem. The presence of the SpoT amplicon is indicated by a black arrow. Glu and rbcL indicated PCR amplicon of plant DNA, used as extraction controls.
